# ConMonity: An IoT-Enabled LoRa/LTE-M Platform for Multimodal, Real-Time Monitoring of Concrete Curing in Construction Environments

**DOI:** 10.3390/s26010014

**Published:** 2025-12-19

**Authors:** Ivars Namatēvs, Gatis Gaigals, Kaspars Ozols

**Affiliations:** Institute of Electronics and Computer Science, 14 Dzerbenes St., LV-1006 Riga, Latvia; gatis.gaigals@edi.lv (G.G.); kaspars.ozols@edi.lv (K.O.)

**Keywords:** Internet of Things, wireless sensor networks, multimodal sensing, concrete curing monitoring, LoRa/LTE connectivity

## Abstract

Monitoring the curing process of concrete remains a challenging and critical aspect of modern construction, often hindered by labour-intensive, invasive, and inflexible methods. The primary aim of this study is to develop an integrated IoT-enabled platform for automated, real-time monitoring of concrete curing, using a combination of LoRa-based sensor networks and an LTE-M backhaul. The resulting ConMonity system employs embedded multi-sensor nodes—capable of measuring strain, temperature, and humidity–connected via an energy-efficient, TDMA-based LoRa wireless protocol to an LTE-M gateway with cloud-based management and analytics. By employing a robust architecture with battery-powered embedded nodes and adaptive firmware, ConMonity enables multi-modal, multi-site assessments and demonstrates stable, autonomous operation over multi-modal, multi-site assessment and demonstrates stable, autonomous operation over multi-month field deployments. Measured data are transmitted in a compact binary MQTT format, optimising cellular bandwidth and allowing secure, remote access via a dedicated mobile application. Operation in laboratory construction environments indicates that ConMonity outperforms conventional and earlier wireless monitoring systems in scalability and automation, delivering actionable real-time data and proactive alerts. The platform establishes a foundation for intelligent, scalable, and cost-effective monitoring of concrete curing, with future work focused on extending sensor modalities and enhancing resilience under diverse site conditions.

## 1. Introduction

Ensuring rapid and cost-effective construction requires effective tools to monitor and control structural integrity and quality. Sensors—particularly wireless sensors and wireless sensor networks (WSNs)—are at the forefront of technological advances in construction, enabling real-time data acquisition and seamless integration that significantly improves management processes in the construction industry. WSNs are especially attractive for construction projects as they enable continuous remote monitoring without the constraints of physical wiring, thereby reducing installation time and costs while enhancing flexibility and scalability [1]. These networks have been widely adopted for applications such as automated construction progress monitoring [2], dynamic construction management systems [3], resource tracking [4], and real-time analysis of worker behaviour [5]. An important subset of WSN applications focuses on monitoring and evaluating the structural integrity of buildings during construction and throughout a structure’s service life [6,7,8,9,10,11]. Such monitoring is essential not only to safeguard investments but also to ensure public safety [12].

A variety of sensors are used in building monitoring, including temperature [13], humidity [14], acoustic [15], acceleration [16], and strain sensors [17]. This diverse range of sensor types enables comprehensive and intelligent building monitoring, highlighting the importance of integrating multiple sensor technologies for more effective data collection and smart building management [18,19]. Among these, vibrating wire sensors (VWSs)—a class of electromechanical sensors—are distinguished by their long-term reliability, stability, and suitability for critical infrastructure measurements [20]. As highlighted in [21], “vibrating wire instruments can provide extremely high levels of stability, accuracy, and durability.”

Despite advances in WSNs and sensor technologies, monitoring the early-stage behaviour of concrete during curing remains a major challenge [22]. Traditional methods for assessing concrete strength, such as laboratory-based cylinder testing, are invasive, time-consuming, and rarely provide the real-time data required for rapid decision-making on construction sites [23]. Because the quality of curing directly affects durability and safety, project stakeholders require timely insights to optimise conditions and mitigate the risk of structural defects.

Recent advances in the Internet of Things (IoT) [24] and low-power wide-area networks (LPWANs) [25]—including long-range (LoRa) and long-term evolution (LTE-M) technologies [26]—offer cost-effective, scalable, and energy-efficient solutions for continuous real-time sensor data transmission. Several studies have demonstrated the use of LoRa or LoRaWAN in wireless underground sensor networks and subsurface environment monitoring, confirming the feasibility of long-range, low power communication in highly attenuating media such as soil and buried structures. However, these systems typically address generic WUSN scenarios rather than embedded concrete curing monitoring, and often assume different MAC protocols, gateway capabilities, and deployment constraints from those found on active construction sites.

Existing approaches to concrete monitoring still face significant limitations. Wi-Fi- or Bluetooth-based systems have short transmission ranges and high energy consumption, while wired solutions involve high installation costs and limited flexibility. Furthermore, few existing systems provide continuous and automated monitoring throughout the critical curing phase, limiting their ability to support timely decision-making.

Several approaches have been proposed for integrating sensors into IoT and WSN platforms for construction and material monitoring. Previous studies have demonstrated such integration across a range of construction and material monitoring tasks, including environmental sensing [27], real-time construction site monitoring [28], structural health surveillance [29], and improvements in IoT for construction safety and efficiency [30]. However, existing methods seldom provide continuous and automated monitoring of concrete during its critical curing phase, thereby limiting their effectiveness in supporting timely decision-making during construction.

To address these limitations, we present ConMonity, an IoT-enabled platform specifically designed for real-time monitoring of the concrete curing process using LoRa and LTE networks. Unlike traditional approaches, ConMonity enables non-invasive, scalable, and continuous data collection by integrating multiple embedded sensors with a gateway-based transmission architecture. This provides construction managers with real-time, data-driven decision support. To the best of our knowledge, this study presents an IoT-based platform for concrete curing monitoring that combines a custom TDMA LoRa sensor network with an LTE-M backhaul, designed specifically for embedded, long-term concrete deployments under realistic construction site constraints.

This work advances existing WUSN and LoRaWAN-based monitoring approaches by providing a concrete-specific, end-to-end design and a field- validated implementation for multimodal, long-term monitoring of concrete curing.

In addition, to positioning ConMonity within the academic literature on WUSN and LoRa/LoRaWAN-based monitoring, the paper provides a structured comparison with representative commercial systems (Giatec SmartRock, Maturix), focusing on sensing capabilities, communication architecture, deployment depth and operational constraints.

The remainder of this paper is organised as follows. Section 2 presents the requirements, design principles, and methodological framework. Section 3 reviews related work and positions ConMonity within the context of existing research. Section 4 describes the validation setup and implementation. Section 5 discusses the results, highlighting the advantages of the proposed platform. Finally, Section 6 concludes the paper and outlines directions for future research.

## 2. Methodology

Building on the research gap identified in the introduction, this section outlines the theoretical foundations and methodological framework employed in the development of ConMonity monitoring platform. It begins by defining the platform requirements, followed by an overview of its core functions and design choices. Subsequently, it describes the research methodology underpinning the platform’s development and concludes by outlining the scope and focus of this study.

The principal objective of this research is to develop a novel automated platform for monitoring embedded concrete curing parameters (strain, temperature, and humidity) during construction phase. The platform specifically targets key functional parameters of concrete, including mechanical performance, curing dynamics, and early-stage crack formation.

### 2.1. System Requirements

To achieve these objectives, the platform was designed in accordance with the following requirements:

- Embedded sensing: Primary data are acquired via vibrating wire strain (VWS) sensors, together with temperature and humidity nodes, which are embedded directly within the concrete matrix.
- Robustness: The system must remain durable and reliable under harsh construction site conditions.
- Real-time data transmission: Continuous wireless communication and timely alerts are essential.
- Energy efficiency: Configurable measurement schedules must optimise power consumption and extend operational life, as embedded batteries cannot be replaced.
- Ease of deployment: Sensor installation must be simple enough for non-specialist personnel (for example, construction workers) without compromising data quality or integrity.
- Low maintenance: The system should operate throughout the construction period with minimal intervention.

### 2.2. System Functions and Design Principles

In accordance with these requirements, ConMonity was designed to perform four core functions:

1. Acquisition of data from embedded VWS sensors, as well as temperature and humidity sensors.
2. Transmission of sensor data to a server-side database and a human-readable interface.
3. Generation of real-time alerts when threshold values are exceeded.
4. Provision of a user-friendly interface accessible to personnel without ICT expertise.

To ensure scalability and accessibility, two additional design principles were adopted:

- Commercial-off-the-self (COTS) hardware: Widely available and cost-effective components were selected to support replicability and affordability.
- Model accessibility: The user interface was implemented as an Android smartphone application, eliminating the need for specialised hardware.
- While the individual technologies used in ConMonity (for example, LoRA modulation, LTE-M connectivity, and MQTT messaging) are well established in IoT practice, their integration into a platform tailored for embedded concrete curing monitoring is a key contribution of this work. The system architecture, link-budget analysis, and TDMA scheduling are jointly optimised for nodes encased in concrete, long-term autonomous operation, and maintenance workflows.

### 2.3. Research and Design Methodology Framework

The development of ConMonity was guided by a comprehensive review of the relevant literature across four thematic domains, each addressing a specific set of implementation-related challenges:

- Network architecture: The optimal monitoring system architecture was examined, including the types and roles of network units, their configuration and interconnectivity, and strategies for effective node management. The key questions addressed were: What should the monitoring system architecture be? Which network units are required? How should connectivity and configuration be defined? How should node management be implemented?
- Sensor connectivity: Various modulation techniques, communication protocols, and medium access control (MAC) schemes were reviewed, along with strategies for implementing time synchronisation. The selection of appropriate antennas for the sensors was also considered. The key questions addressed were: Which modulation and communication protocol should be used? Which MAC scheme is most suitable? How should time synchronisation be achieved? What type of antenna is optimal for sensor nodes?
- Data acquisition and representation: The flow of data for both sensor measurement and network control was defined, and the representation of data at different stages of storage and transmission was examined. The key questions addressed were: What is the optimal path for sensor data acquisition and network control? How should data be represented at each storage and transmission stage?
- Sensor measurement parameters: The physical and environmental parameters requiring monitoring were identified, while suitable measurement methods and instrumentation were evaluated to ensure reliability and accuracy. The key questions addressed were: Which parameters must be measured? Which methods and instruments ensure accurate and reliable measurement?

### 2.4. Research Scope and Focus Areas

The research scope encompasses several key focus areas that define the technical and methodological framework for developing ConMonity monitoring platform:

- Analysis of network structure, connectivity type, data flow, relevant network units and comparable monitoring systems.
- Theoretical calculation of the radio link budget between sensor nodes and the gateway.
- Review of literature on vibrating wire sensors (VWSs), humidity sensors, and related instrumentation, with emphasis on findings most relevant to practical deployment.

Other aspects of system development, such as printed circuit board (PCB) layout and detailed software development, are considered as standard engineering tasks and are therefore not addressed in this paper.

## 3. Related Work

### 3.1. Networks

**Figure 1:**
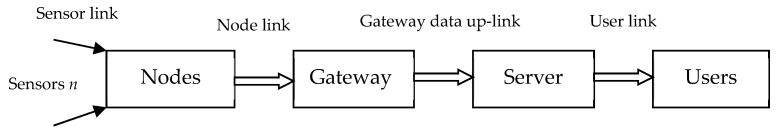
Typical data flow architecture of application-specific sensor networks for construction monitoring.

To identify an optimal network architecture and its constituent components, a comprehensive survey of application-specific sensor networks (SNs) and wireless sensor networks (WSNs) was undertaken. Particular attention was given to monitoring applications with operational requirements analogous to those encountered in construction site environments. These include systems for structural safety monitoring [10,31,32], ground condition tracking [33,34], indoor climate control [35,36], shallow geothermal system monitoring [37], bridge integrity assessment [38,39], rockfall and landslide detection [40], concrete hardening monitoring [41,42], and global maritime information systems [43]. The analysis of these examples reveals common architectural patterns along and domain-specific adaptations tailored to the operational demands of each application. Figure 1 presents a visual summary of the principal network architecture and highlights the key modifications driven by application requirements.

This block diagram highlights a high-level view if sensor network data flow and represents the most widely adopted architecture in building and construction monitoring systems. Multiple sensors (such as VWS, temperature, and humidity sensors) are connected to intermediary nodes. These intermediary nodes relay data to a central gateway, which aggregates incoming measurements. The gateway forwards data to a server (cloud-based or local), which processes and stores the information. End users (for example, construction managers and other stakeholders) access the data via a human-readable interface, such as a dashboard or mobile application. Each segment is labelled, with arrows indicating the direction of data flow.

### 3.2. Sensor Node Link

In wireless sensor networks (WSNs), the sensor node link refers to the communication pathway that enables sensor nodes to transmit collected data to a gateway or central server. Data transmission is typically accomplished using wireless technologies, such as radio frequency or specialised low-power protocols like Zigbee, LoRa, Bluetooth, or various proprietary standards. The selected protocol significantly influences network performance, transmission range, and energy efficiency. Each sensor node is usually equipped with one or more sensors and relies on the node link to forward its collected information across the network [44].

Depending on network topology, environmental conditions, and node placement, connections may be established either directly or by means of multi-hop routing [45]. Multi-hop routing is particularly important in WSNs for improving energy efficiency and reliability, as it enables distant nodes to forward data through intermediate nodes. The performance of the node link is crucial for reliable data transmission across varying distances and under challenging conditions, such as construction sites or underground environments. Key factors, including electromagnetic interference, signal attenuation, and energy consumption, must be carefully controlled and managed to ensure optimal operation.

Advanced modulation techniques, such as LoRa and Zigbee, are widely used, offering high sensitivity, extended communication ranges, and resilience against obstacles or moisture [46]. This resilience refers to the ability of a wireless protocol (for example, LoRa or Zigbee) to maintain signal integrity and reliable data transmission even when physical barriers—such as walls and building materials—or environmental factors—such as humidity, rain, and fog—are present. For instance, LoRa uses chirp spread spectrum (CSS) modulation and operates on lower frequencies, which confers superior resistance to signal absorption and scattering caused by structures such as masonry and concrete. This enables penetration through obstacles and long-range connectivity even in underground and multi-floor environments. LoRa provides long-range transmission and strong obstacle penetration, albeit at the cost of lower data rates, making it more suitable for sparse, wide-area, or underground application (typically up to kilometers and tens of meters for underground deployment) [47]. In contrast, Zigbee typically operates at higher frequencies and with shorter range, and is more susceptible to attenuation from physical barriers and humidity. Zigbee therefore best suited for short-range, densely cluttered indoor environments, supporting mesh networking with higher throughput over short distances, which is advantageous within buildings, but less effective in deeply buried or remote settings [48]. The distinction between LoRa’s long-range capabilities and Zigbee’s short-range mesh performance directly affects the efficiency, scalability, and real-time responsiveness of WSNs design, underscoring the need to align protocol choice with environment- and application-specific requirements, as summarized in Table 1. 

Studies on Wireless Underground Sensor Networks (WUSNs) have attracted considerable research attention, as these systems face signal propagation effects comparable to those encountered in liquid concrete, thereby presenting significant challenges for reliable communication. Bogena et al. (2009) [50] evaluated the performance of a Zigbee-based WUSN, while Dong et al. (2013) [51] developed theoretical models for radio links operating in the 300–900 MHz range and conducted field validation using Mica2 motes. Sun & Akyildiz (2010) [52] propose the use of low-frequency (10 MHz), low-bandwidth (2 kHz) magnetic induction waveguides to enhance signal transmission in moist soil environments.

Collectively, these studies demonstrate that conventional modulation techniques such as Zigbee and magnetic induction, are limited to effective transmission distances of only 3–10 m due to sever signal attenuation in humid environments. For instance, Bracciale et al. (2018) [53] reported that the CC430F5137 wireless MCU chip achieved a receiver sensitivity of −117 dBm at 600 bps, whereas LoRa technology references a sensitivity of −130 dBm at 782 bps.

More recent investigations indicate LoRa and LoRaWAN are promising alternatives for WUSNs, offering significantly improved sensitivity and extended communication ranges relative to other conventional short-range modulation protocols [54]. Hardie et al., 2019 [55] reports that 433 MHz LoRa-based UG2UG operating distances reach 4–20 m, depending on ground type, and referenced −130 dBm sensitivity at 782 bps. Many older WUSN implementations are impractical for underground or concrete environments, which is why newer LPWAN protocols such as LoRa are currently favoured for such demanding deployments, whereas Zigbee or similar hardware rarely exceeds 10 m underground.

Further evidence can be found in [56], which simulates LoRa-based connections and reports a network coverage of several kilometers for nodes buried less than 70 cm. The study also notes that a LoRa-based WUSN node at a burial depth of 50 cm can operate up to 100% volumetric water content, although no specific link range is provided in this saturated scenario.

These findings underscore the potential of high-energy signal modulation schemes for deployment in the proposed platform. Currently, suitable modulations include both LoRa and SigFox, each offering low energy consumption appropriate for long-range IoT applications [57,58]. Additionally, both protocols provide strong resistance to attenuation and support high link budgets (typically exceeding 140 dB) [59], enabling reliable long-range communication essential for IoT applications. However, SigFox exhibits several notable limitations, including a maximum uplink payload size of only 12 bytes per message, limited or optional support for message encryption, and dependance on third-party service providers for network connectivity [60]. Collectively, these restrictions render SigFox unsuitable for the planned application requirements. Consequently, LoRa modulation was selected as the signal transmission method for ConMonity system, offering greater flexibility, security, and integration potential.

### 3.3. Link Budget Estimation

A link budget estimation is essential, as it determines whether the transmitter power and antenna gain enable reliable end-to-end radio communication and quantifies the distance over which communication can occur without repeaters. In this work, the link budget analysis is formulated for homogeneous, unreinforced concrete and is used as a precise site-specific predictor. Its purpose is to provide safe parameter margins, which are subsequently verified and refined through field measurements in real structures.

This analysis also informs antenna selection, which is particularly important given the constraints imposed by irregular building geometries. If results indicate that directional antennas are required, platform implementation becomes impractical, as consistent alignment of embedded antennas cannot be ensured. To preserve the integrity and reliability of the communication platform, the design assumptions must therefore be carefully validated, and approaches that may lead to inconsistent or unreliable performance should be avoided.

The received signal power $P_{rx}$ (in dBm) at the receiver input is calculated using the Friis transmission equation, adapted from [61]:

(1) $$P_{rx}=P_{tx}+G_{rx}+G_{tx}-L_{fs}-L_{conc},$$

where $P_{tx}$ is transmitters power (in dB or dBm), $G_{rx}$ and $G_{tx}$ are the receiver and transmitter antenna gains (dB), $L_{fs}$ is free-space path loss (dB), and $L_{conc}$ is attenuation due to concrete (dB). This provides a framework for quantifying effective signal strength after propagation through obstructive materials, allowing systematic evaluation of link feasibility under practical deployment conditions.

Setting $G_{rx}=G_{tx}=0\;dB$, which is valid if using unit-gain (omnidirectional), the receiver sensitivity $S_{min}$ is specified in dBm. For instance, a high-sensitivity LoRa modem at 7.8 kHz bandwidth and spreading factor 12 has a receiver sensitivity of approximately −137 dBm. The module’s transmit power $P_{tx}$ is taken from the manufacturer’s specification (commonly up to 14 dBm).

The free-space path loss $L_{fs}$ at 868 MHz over 100 m is calculated using standard formula in dB for radio signal traveling over a distance in air [62]:

(2) $$L_{fs}=20{log}_{10}(100)+20{log}_{10}(868)+32.45\approx 71\;dB.$$

The first term, $20{log}_{10}(100)$, represents the contribution from the distance (d), where d=100 m. The second term, $20{log}_{10}(868)$, represents the contribution from the frequency (f), where f=868 MHz. The constant 32.45 is conversion factor ensuring that the units (distance in kilometers and frequency in MHz) match and that the results is in decibels.

The maximum permissible channel loss for a wireless link is:

(3) $$L_{conc}<P_{tx}+G_{rx}+G_{tx}-L_{fc}-P_{rx}=14-0+\;71+148=91\;dB.$$

Equation (3), the link budget expresses the balance of transmitter power, antenna gains, and losses. 91 dB is the margin, the link can tolerate losses due to obstacles, attenuation, inference etc., besides the free-space loss.

Since communication takes place in both directions (antenna embedded in concrete ↔ antenna in free space), the total concrete-related losses are expressed as the sum of the absorption losses in concrete and the boundary reflection losses at the concrete-air interface [63]:

(4) $$L_{tot}=L_{cp}+L_{rca},$$

where $L_{tot}$ is the total concrete related loss, $L_{cp}$ (dB) is the concrete path loss (absorption) and $L_{rca}$ (dB) is the boundary reflection loss $L_{rca}$ (dB) at the concrete-air interface. The boundary reflection loss $L_{rca}$ does not depend on concrete thickness, while absorption increases with the depth at which antenna is embedded. Rather than applying a linear relationship (function) to model concrete path loss, a scaling based on embedment depth is adopted to account for the multi-path effect [61]:

(5) $$L_{cp}=S_{lc}+20*log(h),$$

where $S_{lc}$ is the specific loss (signal attenuation) of the concrete (dB/m) and h is the antenna embedment depth (m).

Experimental data provide quantitative estimates for radio signal attenuation $S_{lc}$ in concrete structures. Jiang S. and Georgakopoulos S.V. [64] report measured losses of up to 15 dB per 25 cm in plain concrete at a frequency of 1 GHz, in the absence of reinforcing bar, which corresponds to $S_{lc}=27\;dB/m$. Jin X. and Ali M. [65] studied signal attenuation in concrete by comparing the performance of embedded simple dipoles and PIFA antennas within concrete structures. When two antennas are embedded within saturated concrete structures, experimental measurements demonstrate that transmission loss can reach 38 dB over a distance of 251 mm, corresponding to a specific attenuation coefficient of approximately $S_{lc}=50\;dB/m$. It should be noted that variations in the chemical composition and physical properties of concrete may result in discrepancies between reported results. For subsequent analytical calculations and design robustness, the upper bound of measured attenuation $S_{lc}=50\;dB/m$ is adopted in subsequent calculations.

Jin X. and Ali M. [65] also measured electrical parameters of saturated concrete: the real part of the dielectric constant $\epsilon_{c}=8.2$, the conductivity σ=0.005−0.05 S/m, and the loss tangent (0.0218–0.1198). Since the conductivity is relatively low (≤0.1 S/m), the boundary reflection loss $L_{rca}$ may be derived from the Fresnel equation [66]:

(6) $$L_{rca}=-10*log(1-{\left(\epsilon_{a}-\epsilon_{c}\right)}^{2}/({\epsilon_{a}+\epsilon_{c})}^{2}),$$

where $\epsilon_{a}$ and $\epsilon_{c}$ are the real parts of the dielectric constants of air and concrete. In the most unfavourable case (saturated concrete), $L_{rca}=4.12\;dB$.

Combining Equations (3)–(6) and solving for maximum penetration depth h under saturated concrete conditions yields permissible depths on the order of a few tens of centimeters for the adopted design margins, indicating that an embedment depth of 0.2 m is well within the available link budget.

(7) $$h<10^{\frac{L_{cp}-L_{rca}-S_{lc}}{20}}.$$

Employing LoRa modulation with a spreading factor of 8 and a bandwidth of 500 kHz lowers the receiver sensitivity threshold ${(P}_{rx}=-126\;dBm)$, while enabling higher data transmission rates of up to 12.5 kbps [67]. This configuration reduces transmission duration, thereby extending the battery life of sensor nodes. However, it limits the effective communication range to approximately 5.55 m. Under burial conditions of 30 cm, the received signal strength remains 25 dB above the receiver threshold, providing a substantial operational margin. This surplus allows transmission power to be reduced from 14 dBm to −3 dBm, particularly when using the RN2483A7 module [68], which further enhances longevity [61]. Consequently, the analytical link budget not only supports but exceeds the requirements for the intended embedment depths of 0.2–0.3 m used in ConMonity.

Although high-gain antennas play a significant role in modern communication systems by ensuring sufficient signal levels for reliable connectivity [69], the link budget calculations for this deployment assume an antenna gain of 0 dB. This approach allows for the use of non-directive (omnidirectional) antennas, enabling construction personnel to embed sensor nodes at any convenient location within the structure. LoRa modulation thus facilitates the practical application of such non-directive antennas in building environments [62].

Furthermore, with the received signal strength (RSS) exceeding the receiver’s sensitivity threshold, the radio link can be reliably established even if additional losses occur in the communication channel [70]. These losses may arise from less efficient antennas or obstructions such as construction equipment along the path of the radio link, but the maintained signal margin ensures robust node connectivity.

### 3.4. Data Transfer Protocol and Media Access

LoRa communication is frequently implemented using the LoRaWAN protocol, a standardised and widely adopted framework designed for long-range, low-power wireless networking applications [71,72,73,74,75,76,77,78,79]. However, to preserve the cost-effectiveness of the monitoring platform, standard LoRa gateways—typically associated with higher costs—are intentionally excluded from the system architecture. Instead, both the gateway and the sensor nodes integrate a single LoRa radio module configured to operate on a fixed frequency channel, thereby enabling direct point-to-point or peer-to-peer connectivity [80]. This simplified architecture reduces infrastructure costs and maintains reliable data transfer, making it particularly suitable when a full LoRaWAN network deployment is not required.

For media access control, Time Division Multiple Access (TDMA) is required. However, native LoRaWAN specifications make TDMA impractical in high-density deployments. The mandatory delays (such as JOIN_ACCEPT_DELAY1 = 5 s, JOIN_ACCEPT_DELAY2 = 6 s, and RETRANSMIT_TIMEOUT = 1–3 s) [70] severely limit timing granularity. For instance, in a platform with 250 nodes and a TDMA frame of 15 min, each node would only be allocated only a 3.6-s transmission slot, which is incompatible with LoRaWAN timing constraints. To address this issue, two strategies are possible [81]:

- Reduce the number of nodes to keep slot length within feasible limits.
- Develop a customised non-LoRaWAN TDMA protocol tailored to the monitoring system requirements.

For this reason, the platform does not use the LoRaWAN network protocol. Instead, it implements a custom TDMA-based MAC over raw LoRa physical-layer links.

In the latter case, precise time synchronisation is crucial to avoid packet collision. In Ref. [82], a lightweight synchronisation method that uses broadcast messages embedded within network state updates is proposed. Alternatively, Ref. [83] presents a more advanced scheme requiring a LoRa gateway capable of receiving multiple modulation parameters simultaneously—a capability not supported by single-radio configurations.

Another challenge arises from clock drift in battery-powered nodes, particularly as supply voltage fluctuates or decreases over time. This affects oscillator accuracy and leads to timing errors, causing nodes to miss regular synchronisation messages and adversely affecting protocols such as TDMA, which require precise timing coordination [84]. This undermines the reliability of lightweight synchronisation protocols such as Ref. [85]. Over long-term deployments, maintaining synchronised TDMA scheduling becomes increasingly difficult in energy-constrained environments. Maintaining long-term synchronisation is especially difficult in energy-constrained environments, where frequent re-synchronisation would reduce battery life and lightweight protocols may lack robustness to drift. This limitation highlights a key trade-off between energy efficiency and synchronisation accuracy, which may necessitate [86]:

- Incorporate more robust synchronisation mechanisms, including redundancy and clock correction algorithms.
- Adaption hybrid access schemes that combine content- and schedule-driven methods.
- Developing adaptive protocols capable of tolerating occasional clock drift or missed slots without leading to network-wide communication failures.

As an alternative approach, a master node (gateway) that remains constantly active is deployed [87]. In this configuration, the gateway processes all incoming packets and transmits accurate time information within the acknowledgement message during each node’s transmission slot. For reliable TDMA operation, the duration of each timeslot must be long enough to accommodate clock drift between successive transmissions. To prevent slot overlaps, the drift must remain smaller than the designed guard interval between slots.

In scenarios where nodes transmit only once per day, accumulated clock drift may reach several seconds, necessitating longer TDMA timeslots to ensure collision-free operation. To maintain reliability, timeslots of ten seconds are commonly adopted, ensuring that the drift remains smaller than the guard interval between adjacent slots [88]. A longer time slot also enables bidirectional communication: after transmitting a measurement packet, the node may receive configuration updates or correction commands from the gateway within the same communication window. This capability supports adaptive system behaviour, remote parameter tuning, and improved manageability of the network in long-term deployments.

### 3.5. Data and Node Management

Initial design efforts focused on key aspects such as the data acquisition pathway, storage and representation formats, node control logic, and message protocols. However, it was observed that the published scientific literature frequently lacks detailed information regarding practical implementation details, especially for real-world applications [89].

The data flow architecture must confirm to the structure outlined in Figure 1, ensuring seamless integration between sensors and the server platform. The node control logic must support dynamic merging, allowing new nodes to automatically synchronise and operate alongside an existing network [90]. Conversely, the system must also provide mechanisms for the reliable removal of malfunctioning nodes, including those affected by power depletion or battery failure.

In addition, the monitoring system should include node management functions accessible via the user interface [91]. The system accommodates configuration updates originating from either the server or the gateway. These updates include the synchronisation of timestamps, transmission scheduling parameters, and sensor operation settings [92]. Finally, to optimise battery life—particularly in resource-constrained nodes and gateways [93]—the messaging protocols must be designed with minimal payload sizes, ensuring energy-efficient communication without sacrificing reliability or configurability.

### 3.6. Sensors Implementation of Embedded Sensors

Sensors such as vibrating wire sensors (VWSs) or other structural and geotechnical instruments are typically connected to sensor nodes via wired interfaces, ensuring reliable excitation and data acquisition [94]. The monitoring system primarily collects data from VWSs and humidity sensors (HSs). Additionally, monitoring the battery voltage of each node is recommended to estimate the remaining useful operational life and support predictive maintenance.

Each integrated sensor introduces its own implementation-specific requirements, including variations in signal processing, sampling timing, and communication protocols. These aspects must be carefully coordinated within the overall node control and data acquisition architecture.

The VWS represents the most complex sensing element in the monitoring system due to its time-consuming and technically demanding measurement procedure. As documented in [8,10,37], the measurement process requires frequency sampling over a predefined range, with the sensor’s resonant behaviour detected via a pickup coil. Around 500 oscillations are typically required to obtain a reliable measurement. With a resonance frequency near 1000 Hz, this corresponds to a minimum acquisition time of 0.5 s per range. During this process, harmonic components resulting from oscillation outside the fundamental frequency can induce signals in the pickup coil. These harmonics must be addressed in the signal processing stage to ensure accurate resonance detection and measurement fidelity.

**Figure 2:**
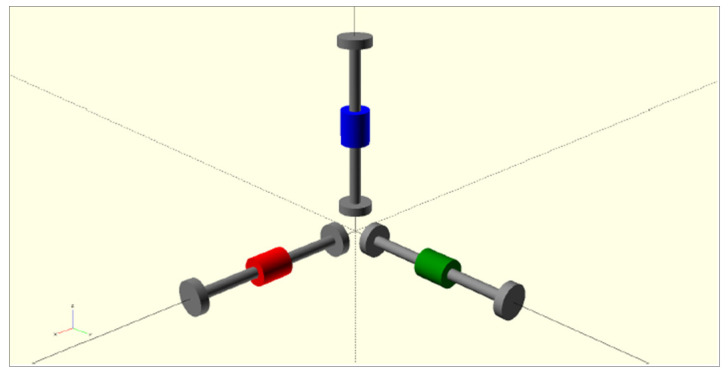
The placement of the strain gauge sensors for the strain measurement in all axes. The three colours indicate the orientation of the embedded strain gauges: the blue element represents the vertical sen-sor, while the red and green elements correspond to the two horizontal sensors aligned with orthogonal axes.

Typically, three VWS are usually installed along the main axis of the monitored structure, see Figure 2.

To reduce overall costs, each sensor node should therefore support multiple VWS channels, enabling a single node to interface with several sensors. This reduces hardware redundancy and streamlines system development.

Manufacturers and previous studies [9,37] highlight the importance of temperature compensation in VWS measurements. Since resonance frequency is sensitive to temperature fluctuations, VWS units typically include an integrated thermocouple or thermistor. Consequently, the sensor node must be capable of interfacing with and acquiring data from both sensor types to achieve temperature-compensated strain measurements under varying environmental conditions.

The humidity sensor presents a different challenge: ensuring long-term reliability under extreme conditions. Environments such as fresh concrete exhibit 100% humidity and highly alkaline, ion-rich, chemically aggressive conditions, which can destroy conventional moisture sensors in less than 72 h. To address this, the sensor must be housed in a protective yet permeable enclosure. This design allows vapour diffusion while shielding the sensing element from corrosive exposure, significantly extending its operational lifetime in cementitious environments.

Finally, battery voltage monitoring must be implemented in an energy-efficient manner. Rather than relying on a resistive voltage divider, which continuously consumes current, the microcontroller can measure its internal reference voltage against the battery supply. This indirect method allows periodic assessment of battery status with minimal energy overhead—a widely adopted technique in low-power embedded systems.

## 4. Results and Evaluation

### 4.1. ConMonity Platform

**Figure 3:**
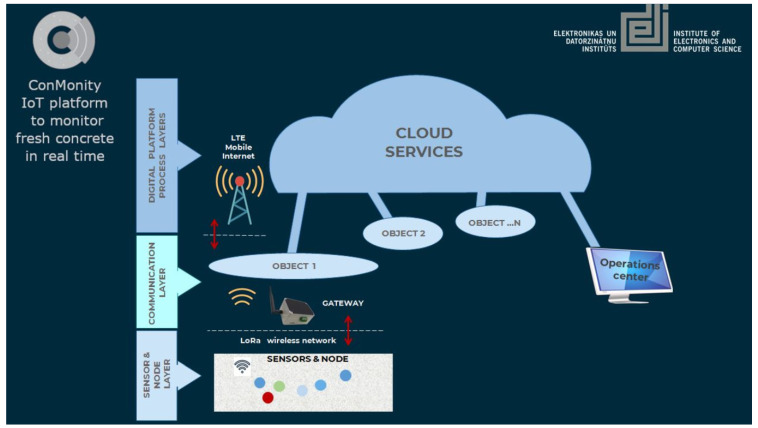
ConMonity IoT platform architecture for concrete monitoring in real time.

ConMonity system architecture (Figure 3) reflects contemporary wireless sensor network (WSN) for infrastructure monitoring. Sensors—including strain gauges, humidity, and temperature sensors—are connected via wired interfaces to sensor nodes, which transmits the measured values to a gateway via the LoRa protocol. The gateway collects readings from up to 256 nodes and forwards all data to a cloud-based server/database via MQTT. The selected database is optimized for MQTT integration and server-side Python 3.8 scripting, enabling seamless processing and direct storage of incoming data streams.

User access is provided through a dedicated Android OS application, which supports real-time data visualisation and ensures that users receive automatic alerts even when the application is inactive. This design supports the simultaneous monitoring of multiple structures and sites, enabling remote supervision of curing processes, minimising transport costs, and reducing personnel workload. Automatic alerting, triggered by predefined system rules, enhance ease of use and system responsiveness. The principal functional parameters of ConMonity platform are summarized in Table 2.

The results of the implementation are presented from the bottom up and reflect the hierarchical integration of sensors, nodes, gateways and remote control interface components.

### 4.2. Sensors

The performance of ConMonity system is determined by the careful selection and integration of high-quality sensing elements capable of delivering accurate, stable, and long-term measurements under demanding conditions within embedded concrete structures. Each monitoring node, deployed to supervised concrete curing, is equipped with three strain gauge sensors with integrated temperature measurement and dedicated humidity sensor.

For reliable tension readings from vibrating string sensors, it is essential to account for both the natural frequency and temperature during calculation. This requirement necessities positioning the temperature sensor in close proximity to the vibrating string sensor. Accordingly, the sensor housing incorporates accommodates both components in adjacent positions. For energy efficiency, each node monitors the state of charge of its battery prior to data transmission, thereby conserving power and enhancing system longevity by minimizing communication failures due to the depleted energy reserves.

Strain gauge sensors: During the development phase of ConMonity, two vibrating wire strain sensor models—Model 4200 (GEOKON) [74] and BM150 (BEWIS SENSING) [75]—were systematically evaluated for their suitability in monitoring strain through the concrete curing process.

- *Model 4200:* This sensor integrates a vibrating wire element with a thermistor, enabling temperature compensation and measurement of strain via changes in wire vibration frequency induced by concrete deformation. This dual sensor configuration ensures robust and accurate strain readings under variable thermal conditions and conforms to the best practice in embedded strain monitoring [95].
- *BM150:* Similar to the Model 4200, the BM150 uses vibrating wire technology features a short gauge length (100 mm compared to 150 mm for Model 4200), which results in a lower amplitude response likely due to the reduced measurement span. Experimental tests confirmed that both sensors can be driven to resonance by rectangular signal pulses, facilitating efficient strain detection [96].

Sensor performance evaluation focused on temperature compensation, signal noise reduction, and measurement stability, which are essential for effective monitoring using vibrating wire technology. Both tested sensors experienced challenges relating to energy transfer from the excitation coil and inconsistent mechanical resonance detection, issues which can hinder the accurate identification of resonant frequency and thereby affect measurement precision. Nevertheless, optimisations were implemented in the measurement algorithms, such as triggering frequency range scanning only upon significant deviations from baseline readings, resulting in more rapid and efficient data collection. This approach is consistent with contemporary sensor signal processing strategies, aiming to reduce the impact of ambient noise and enhance the reliability of long-term structural health monitoring.

Temperature measurement: Both strain gauge models incorporate thermistors for temperature measurement, enabling accurate compensation of reading during dynamic concrete curing. The node’s input design offers flexibility, allowing the selection between thermistors and thermocouples via appropriate divider resistor values. To ensure data integrity, temperature is measured using a single-ended configuration with low-pass filtering to ensure stability, reducing signal noise and enhancing the precision of acquired data—each of which is critical for compensating string measurement errors caused by temperature fluctuations during concrete curing.

**Figure 4:**
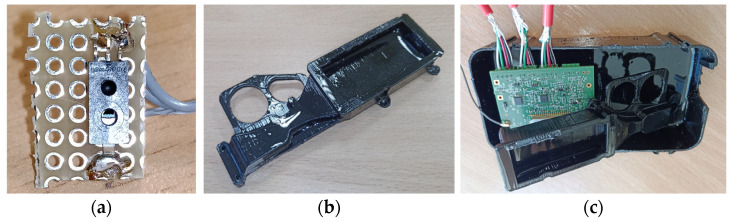
(**a**) the humidity sensor HIH-5030-00, (**b**) the protective chamber, (**c**) humidity sensor chamber and node circuit board before mounting in the node enclosure.

Humidity sensor: Long-term measurement of humidity in concrete is notoriously difficult. Initial tests showed that the sensors often failed when exposed to fresh concrete [97], resulting in unreliable data and a loss of confidence among site personnel. Fresh concrete exhibits pore solution pH values in the range 12.5–13.9, which are known to cause stability and lifetime issues for many embedded electromechanical and fibre-optic sensors unless they are specifically adapted for this environment. These sensors often require modification or protective layers, as they are otherwise damaged or drift when exposed directly to fresh concrete pore solution [98]. A Honeywell HIH-5030-001 humidity sensor was selected for its low current consumption (500 μA). To enable effective humidity measurements, the sensor was isolated from direct contact with the concrete by enclosing it within a specialised housing, allowing humidity exchange with the concrete via a porous ceramic filter and ensuring suitability for high-pressure immersion (see Figure 4).

The humidity sensor used in this study is a capacitive-type device with overall dimensions of 2.67 × 8.59 × 4.17 mm. It operates over a range from −40 to +85 °C and supports a supply voltage between 2.7 V and 5.5 V. The specified measurement accuracy is ±3%.

This approach achieves several objectives: it physically isolates the humidity sensor from wet concrete prevent degradation, minimises condensation effects by means of tight encapsulation, and ensures consistent and accurate readings regardless of sensor orientation. These factors are essential to maintain long-term reliability in embedded concrete monitoring systems.

### 4.3. Sensor Node Hardware

The hardware architecture of each sensor node forms the foundation of ConMonity monitoring system, integrating durable wireless communication capabilities, an optimised antenna setup, and robust environmental protection. These design elements collectively ensure that each node delivers reliable, real-time performance in demanding embedded structural settings, supporting accurate and continuous monitoring in concrete environments. The hardware architecture features strong wireless each sensor node in ConMonity monitoring system features strong wireless communication components, a carefully optimised antenna configuration, and comprehensive environmental protection measures to ensure dependable operation.

**Figure 5:**
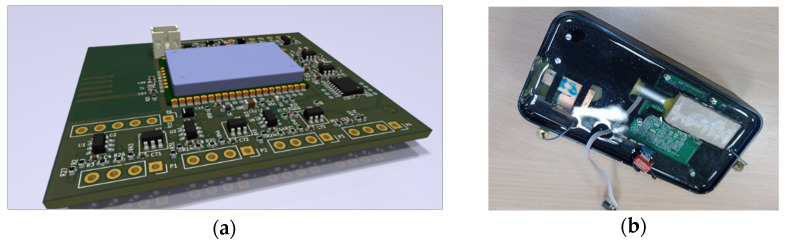
(**a**) the final design of the sensor node PCB, (**b**) antenna testing.

Communication module: At the core of each sensor node is Microchip RN2483A module, selected for its integrated LoRaWAN protocol stack and full compliance with European (EC) and US (FCC) regulatory standards. Operating in the 868 MHz ISM band, the RN2483A supports data rates from 0.3 to 50 kbps and provides a link budget exceeding 157 dB. This enables a communication range of several kilometers in rural areas and 2–5 km in urban contexts, given appropriate antenna matching. The module ensures data confidentiality and integrity through integrated AES encryption, while a low-power sleep mode (< 1 µA) makes it highly suitable for long-duration, battery-powered deployments. A three-dimensional rendering of the finalised printed circuit board (PCB) design is provided in (see Figure 5).

Comparative integration: Alternative LoRa solutions, such as the Semetech SX127x or HopeRF RFM95, offer similar radio performance but require considerable effort for certification and manual protocol stack integration. In contrast, the RN2483A provides a pre-certified, integrated solution that reduces complexity and deployment costs, making it ideal for prototyping and rapid field adoption.

Antenna design: Each node employs a compact Planar Inverted-F Antenna (PIFA), integrated onto the PCB and tuned for 868 MHz operation. The antenna design compensates for the dielectric properties of concrete, accounting for a typical relative permittivity $(\varepsilon_{r}\approx 4-5$) that increases during hydration. PIFA antennas are recognized for maintaining stable performance when embedded, outperforming standard dipole configurations in such environments, and provide cost-effective system scalability.

**Figure 6:**
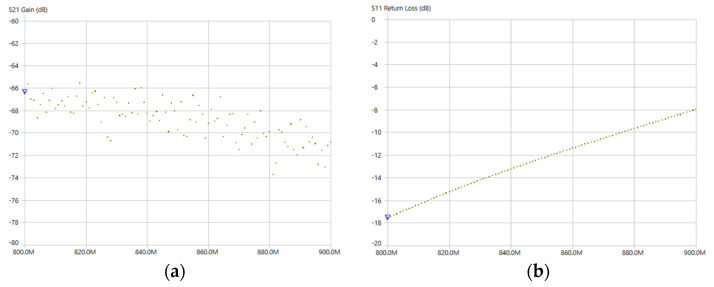
(**a**) Measured forward transmission coefficient S21 gain of the embedded PIFA antenna-concrete setup over the 800–900 MHz range. (**b**) measured input refection coefficient S11 over the 800–900 MHz frequency range.

To verify the RF behaviour of the embedded PIFA and support the link budget assumptions, the antenna-concrete configuration was characterized using a vector network analyser. Figure 6a presents the measured S21 forward transmission coefficient, which remains in the range from approximately −66 dB to −75 dB across the LoRa operating band around 868 MHz, indicating a relatively flat frequency response within the band of interest and confirming that the encapsulated, embedded antenna maintains sufficient forward transmission for reliable sub-GHz LoRa communication through concrete.

The input matching of the antenna-sensor interface was assessed via the input reflection coefficient (return loss) S11 over the same frequency interval, as shown in Figure 6b. The return loss is about −18 dB at 800 MHz and increases roughly −8 dB at 900 MHz, staying better than −10 dB for the majority of the operating ranges, which demonstrates an acceptable impedance match and supports efficient power transfer between the RF front-end and the embedded PIFA for the intended deployment conditions.

Environmental protection: To ensure long-term reliability, all electronics are encapsulated in epoxy resin. This design shields the circuit from corrosive effects caused by exposure to fresh concrete, maintains stable electromagnetic performance in a high-dielectric setting, and protects battery function under mechanical and thermal stress. The integrated approach to antenna configuration and full encapsulation comprehensively addresses both RF and durability demands typical in civil engineering applications.

### 4.4. Sensor Node Firmware

The firmware of the sensor node orchestrates sensor data acquisition, secure communication, and efficient energy management, forming a pivotal operational layer for ConMonity system. Build on prior research and the open LARio firmware for the RN2483A module—derived from Microchip’s proprietary codebase—the firmware incorporates a real-time scheduler, extended sensor drivers, and a tailored protocol stack designed specifically for WSNs.

The node firmware was developed specifically for the custom LoRa-based sensor ConMonity platform and is responsible for managing analogue signal acquisition, preprocessing, power-state control, and LoRaWAN communication. The firmware runs on the microcontroller and interfaces with the Microchip RN2483A LoRa transceiver module. During operation, the firmware initialises the analogue front-end, performs multichannel ADC sampling, and applies internal consistency checks before preparing data for transmission. A detailed serial debug log, captured with PuTTY during development, shows the step-by-step execution of the measurement cycle: ADC setup (INITADC4VVC, INITADC4AMP), analogue stabilisation phases (WAIT4VCC, WAIT4Anal), channel iteration (NEXTCH:0), and sample acquisition for each analogue input. The MEMORISE and channel-scanning messages (the firmware cycles through channels) indicate that the firmware iterates over multiple sensor channels (for example, 0, 1, 2, …) stores or “memorises” the most recent measurements, and uses the SKIP_SCAN_IF_OK condition to bypass repeated acquisitions on channels whose signals remain within acceptable bounds (for each channel, it compares signals and may skip repeated scans if values are stable). The repeated INIADC4AMP messages indicate that, for each sensor channel, the firmware invokes the amplifier-channel ADC routine many times in succession, which is consistent with an oversampling or averaging strategy but could also reflect a hardware or configuration issue that forces unnecessary reintialisation cycles.

**Figure 7:**
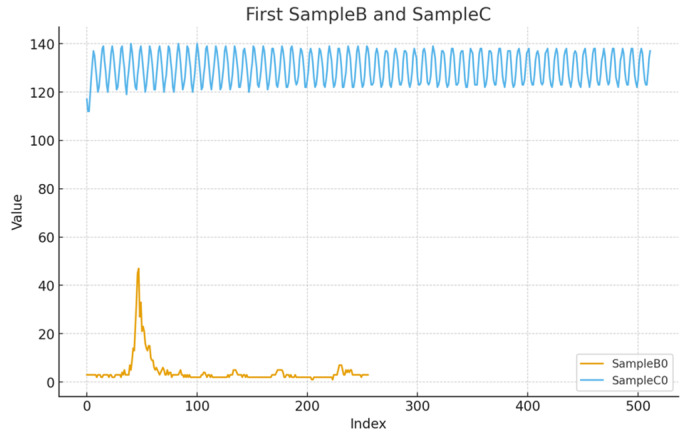
SampleB and SampleC measurements from a single embedded sensor channel.

Figure 7 illustrates the first pair of SampleB and SampleC measurements acquired from a single sensor channel of the embedded node. The horizontal axis represents the same index, while the vertical axis shows the corresponding integer sample value. SampleB (orange) consists of 256 points and exhibits a clear transient response with a peak near index 50, followed by a gradual decay towards a low baseline, indicating a localized excitation captured at low amplitude. SampleC (blue) contains 512 points and forms a nearly stationary oscillatory waveform with values between approximately 120 and 140, representing an amplified or differentially filtered version of the same underlying signal over a longer sampling window.

#### 4.4.1. Real-Time Task Scheduler

A priority-based scheduler manages resource allocation, optimising sensor polling intervals, and minimising energy consumption over extended monitoring periods. Key features include:

- Granularity: Tasks are scheduled with 2-s granularity, and repeat intervals can be configurated from 15 min up to 24 h.
- Predefined operations: “Measure” tasks synchronise sensor data collection across all nodes (observed drift <1 s after hourly synchronisation via gateway acknowledgement). “Transmit” tasks are staggered individually for each node, according to its network ID and schedule settings.
- Task management: Each task concludes with a timeout and returns the node to its lowest power mode to conserve energy.
- Energy optimization: Both the radio and microcontroller subsystems regularly enter deep sleep between duties, extending battery life, and system reliability.

#### 4.4.2. The Protocol Overview

Communication within the network is managed using a custom frame format and checksums to ensure basic integrity for unencrypted transmissions. AES-128 encryption, implemented via Microchip library, is available at compile time to enhance confidentiality when required.

Network Topology: Homogenous sensor nodes join a time division multiple access (TDMA) scheduled network under the gateway master, supporting up to 253 nodes. TDMA ensures organised channel access, minimising collisions and reducing radio-on time.

Node operation and sleep management: Sensor nodes operate with long sleep intervals, waking only to measure and transmit during their assigned window. The gateway remains continuously active for configuration tasks and to process data communication from mis-scheduled nodes.

Node identification and joining: New nodes initialise with ID 0, enabling the gateway to assign unique identifiers (2–254, with 255 reserved). Membership is confirmed via management commands included in gateway.

Acknowledgment and power management: Nodes adaptively adjust transmission power—raising output following missed acknowledgments and lowering it when successful transmissions are confirmed. This dynamic adjustment helps sustain reliable links as concrete properties evolve over time.

Packet structure: There are four principal data packet types (24 bits each)—one for direct data transmission, three for network management, and exchange.

LoRa modulation parameters: Selected LoRa parameters include a spreading factor (SF) of 10, bandwidth (BW) of 250 kHz, baud rate of 1953 bps, implicit header, coding rate 4/5, no CRC, no IQ inversion, and a preamble length of 12.

Time synchronization: Strict TDMA operation requires node clock to be in synchronisation. Gateway acknowledgments include the correct time, enabling nodes to syncchronise their internal clocks and correct for time drift.

Transmission windows: The node join window is between 2–4 s, and the data transmission window allows 10 s per node. This structure supports hourly transmission from all nodes, facilitating timely and scalable sensing cycles.

Data storage and security: Data records are buffered in node EEPROM, ensuring their integrity prior to extraction. Data can be accessed via serial commands when the node is physically retrieved.

The firmware architecture ensures synchronisation, secure, and energy-efficient WSN operation, supporting robust real-time monitoring of concrete curing on a large scale.

### 4.5. Gateway Hardware Selection

The gateway hardware serves as the central aggregation point, maintaining a reliable connection between distributed sensor nodes and the backend infrastructure for real-time concrete monitoring. A dedicated gateway was developed to aggregate sensor data, perform protocol translation, and enable seamless backend integration.

The cellular IoT prototyping platform Nordic Thingy:91 was selected as the gateway controller for its robust performance and global compatibility. Key technical features include FCC (USA) and CE (EU) certifications, integrated LTE-M/NB-IoT/GNSS antennas, external antennas connectors, +23 dBm LTE-M RF output power, and a rechargeable 1350 mAh Li-Po battery. The compact form factor (58.8 mm × 58.5 mm × 19.7 mm, 61 g) and support for eSIM facilitate versatile deployment. Free input/output (I/O) and serial ports enable direct connection to LoRa radio modules.

Bluetooth Low Energy (BLE) and nRF52840 controller connectivity are not utilised in this project. For cost-effectiveness reasons, the gateway employs a single LoRa radio module, which functions as the network master and is programmed via Thingy:91 serial port. The LoRa scheduler is disactivated, with network protocol logic managing the master functions, and power is supplied directly by the main controller.

**Figure 8:**
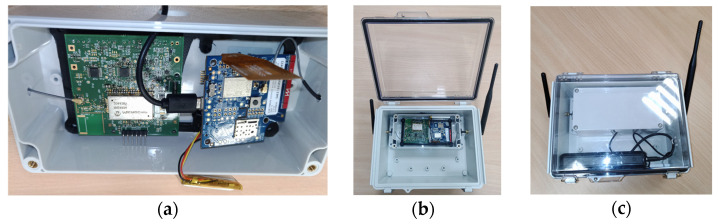
(**a**) gateway electronics, (**b**) gateway antennas placement and electrotonic assembly within enclosure IP68, (**c**) assembled gateway with battery.

During the platform’s integration, particular emphasis was placed on safety and ease of maintenance. All main electronic and radio modules are housed in a tamper-proof casing with an integrated USB port, allowing battery replacement without exposing internal components. The LTE and ISM antennas are positioned to optimise signal coverage. Only authorised access is permitted (see Figure 8).

This multi-layer design ensures that the gateway establishes an uninterrupted, secure connection to all sensor nodes and reliably forwards data to the backend infrastructure, enabling continuous and automated monitoring of concrete curing across varied environments.

### 4.6. Gateway Firmware

The gateway firmware manges the LoRa radio network and LTE cellular connectivity via an industrial SIM card. It receives data from TDMA-scheduled nodes, registers new nodes, distributes network commands, and validates and forward data to the backend server. The gateway hardware incorporates a 20,000 mAh power bank (or 220/12 V adapter), supports LTE bands B2, B4, B5, B12, B66, and B71, is equipped with a 4 G FRP omnidirectional antenna, and includes an integrated LoRa module operating at 868.8 MHz.

The main firmware components include:

- Network the 24/7 active master node management (modem synchronisation, initialisation, command dispatch, packet parsing and routing).
- LoRa-based network management application programming interface (API, packet processing).
- Client-side MQTT procedures (embedding time information into knowledge) ii.

A binary-coded MQTT protocol is used for uplink data transmission, significantly reducing size compared to JSON—an approach that saves battery power and optimises cellular data use. The MQTT client maintains the broker connection, publishes binary data from the sensor validation and error routines, and transmits network management messages. Overall, the firmware ensures efficient network communication management, protocol translation, and minimised power consumption, supporting the sustainable, long-term gateway operation required for continuous, battery-powered deployments.

The field measurements were performed with a custom battery-powered data logger integrating a Microchip RN2483 LoRa transceiver and an embedded omnidirectional antenna. The logger was connected to an embedded vibrating-wire strain gauge with integrated sensors, and, where applicable to additional humidity sensors via shielded cables routed to the node. Power was supplied by two lithium-based cells with a nominal capacity of 20,000 mAh, selected to support multi-month autonomous operation under the configurated duty cycle. During the experiments, the logger firmware executed a fixed measurement timetable in which strain and temperature were sampled every 15 min, and buffered locally before uplink transmission over the LoRa link. Each measurement cycle consisted of sensor excitation, signal acquisition, on-node preprocessing, and packet transmission, followed by a low-power sleep mode until the next 15-min interval.

## 5. Discussion

ConMonity represents a new generation of embedded wireless concrete monitoring systems, integrating LoRa and LTE connectivity, multimodal sensing, and real-time remote access. Compared to established systems such as Giatec SmartRock, Maturix, and other LPWAN-enabled platforms, ConMonity offers several distinctive and practical features.

**Architecture and Connectivity:**

- Wireless protocols: Most commercial and academic systems employs Bluetooth LE, Zigbee, LoRaWAN, or cellular connections for data transmission. Bluetooth LE-based sensors such as Gigatec SmartRock, facilitate convenient data retrieval via smartphones but require site visits and offer limited penetration. LoRa-based solutions, including ConMonity, provide greater range and support deeper embedding in concrete structures.
- Edge-to-cloud data flow: ConMonity uniquely combines a LoRA TDMA-based wireless network for node communication with direct LTE-M/NB-IoT backhaul via the gateway. This hybrid approach enables large-scale, distributed deployments and facilitates flexible backend integration, in contrast to single-protocol systems, which can be limited in range, scalability, or may incur higher subscription costs.

**Sensing Capabilities:**

- Sensor array: Most wireless concrete monitoring solutions primary focus on embedded temperature measurements to estimate maturity and strength, sometimes supplemented by humidity monitoring. ConMonity extends this functionality by supporting the simultaneous measurement of strain, temperature, and humidity at each node, providing a multi-dimensional assessment of the curing process, and the structural health condition.
- Robustness and depth: Fully embedded wireless sensors often have limited signal transmission through thick concrete. Many commercial sensors mitigate this by placing transmitters close to the surface or limiting the depth of deployment. In contrast, ConMonity nodes incorporate encapsulation and advanced power management strategies to maximise battery life (up to 2 years), and ensure reliable data transmission, even when embedded within structural elements.

**Data management and handling.**

- Sensing Capabilities: Earlier wireless concrete monitoring systems often require manual data download or site visits for data retrieval. In contrast, ConMonity supports real-time remote monitoring and automatic alerts via a cloud-connected infrastructure and an Android application, significantly enhancing the responsiveness of site personnel.
- Data integrity and security: While many commercial sensors store data internally for later retrieval, ConMonity’s protocol buffers transmissions in EEPROM to ensure redundancy, transmits binary MQTT payload to minimise cellular data usage, and can employ AES encryption for sensitive deployments, thereby increasing both reliability and efficiency.

**Long-term deployment and scalability.**

- Power management: Like other leading LPWAN-based solutions, ConMonity employs aggressive sleep scheduling and adaptive transmit power control, enabling multi-month autonomous node operation. This supports cost-effective, relative long-term deployments in construction monitoring environments.
- Cost and scalability: Commercial wireless sensors often incur higher initial costs and may require individual cloud subscriptions. By contrast, ConMonity’s system is designed for both academic and industrial scalability, supporting up to 256 nodes per gateway and allowing open integration with backend databases and custom application layers.

**Novelty and positioning**

Existing WUSN and the custom LoRa TDMA network illustrate how LoRa can be used for underground and subsurface monitoring. However, they typically address either generic soil conditions, short-term experiments, or different MAC-layer assumptions. In contrast, ConMonity targets embedded concrete sensors and combines a custom TDMA LoRa network with an LTE-M backhaul, with the protocol design, antenna assumptions, and power budgeting explicitly derived for concrete attenuation, embedment depth, and construction-site operation. The contribution therefore lies in this application-specific integration and engineering, rather than in the mere co-existence of LoRa and LTE technologies.

**Comparison with commercial monitoring systems**

Recent commercial solutions such as Giatec SmartRock and Maturix provide practical tools for monitoring concrete curing. However, their design and capabilities differ in several important aspects from the proposed ConMonity platform. A structured comparison focusing on sensing modalities, communication technologies, deployment depth, autonomy, and data access models reveals these differences. While most commercial systems primarily measure temperature and rely on short-range or infrastructure-dependent communication (for example, Bluetooth, LoRaWAN, NB-IoT), ConMonity supports embedded strain, temperature, and humidity sensing, and uses a custom TDMA-based LoRa link combined with LTE-M backhaul to enable continuous, long-term monitoring of embedded sensors.

It should be noted that detailed proprietary performance and cost data for commercial platforms are only partially available in public sources. Consequently, the comparative analysis focuses on documented features, typical development models, and reported performance indicators, while avoiding quantitative claims that cannot be independently verified. Superiority claims are therefore limited to aspects directly demonstrated in our field measurements (for example, stable communication from nodes embedded 20 cm in concrete, multimodal sensing capability, and sustained autonomous operation over the reported multi-month deployment), or are expressed as advantages in specific dimensions such as sensing richness, deployment depth, and openness of the architecture.

The main characteristics of ConMonity, Giatec SmartRock, and Matrix are summarised in Table 3, emphasising differences in sensing scope, communication architecture, embedding depth, autonomy, and access model.

Despite these advantages, challenges remain for wireless embedded systems, including pronounced attenuation of radio signals in dense concrete, interference affecting humidity sensors, and bandwidth limitations that arise in very high-density installations. Compared to fibre optic or electrochemical sensor platforms, ConMonity prioritises economical, multi-parameter monitoring, and flexible infrastructure over ultra-high precision or specialize analytical functionalities.

**Study Limitations**

The study addresses the design and initial validation of the ConMonity platform during realistic, time-bounded field deployments, focusing on the early and medium-term phases of concrete curing and the related communication and sensing performance. Although the system is designed for long-term autonomous operation, with node lifetimes estimated at up to approximately two years, this manuscript reports only on multi-month deployments and does not include a single, continuous multi-year monitoring campaign. Therefore, the results presented should be interpreted as evidence of feasibility and stable operation during curing and early service period, rather than as full verification of long-term durability.

In addition, the radio link budget and antenna design are based on homogeneous, unreinforced concrete, using upper-bound attenuation values from saturated concrete to ensure conservative design margins. These may not fully represent the behaviour of densely reinforced or highly heterogeneous structures. While the successful field deployment suggest that these conservative assumptions are adequate for the reported scenarios, comprehensive validation across a wider range of structural configurations, admixture types, and thermal conditions remains future work.

## 6. Conclusions

This work introduces ConMonity, a concrete-structure monitoring platform that integrates embedded multi-sensor nodes, a custom LoRa-based wireless sensor network, and an LTE-enabled gateway into a unified architecture for automated assessment of concrete curing and early-age structural behaviour. Field deployments under laboratory construction conditions indicate that the system can provide stable, multimodal measurements of strain, temperature, and humidity from embedded nodes over multi-month periods, supporting the adequacy of the link-budget design, antenna configuration, and power-management strategy for practical site operation.

From an architectural perspective, ConMonity demonstrates that combining TDMA-scheduled LoRa communication with binary MQTT over LTE-M achieves a favourable balance between communication robustness, energy consumption, and cellular bandwidth usage for infrastructure monitoring scenarios. Although the radio modules are LoRaWAN-capable, ConMonity uses a custom LoRa TDMA MAC rather than the LoRAWAN network protocol, to achieve precise time slot scheduling for dense embedded deployments. The modular hardware and firmware stack, based on certified RF modules and extensible sensor interfaces, simplifies regulatory compliance and allows the same platform to be adapted to additional sensing modalities or other structural materials without fundamental redesign.

Comparative analysis with representative commercial systems suggests that ConMonity occupies a distinct position in the design space, prioritising embedded, multi-parameter sensing and open backend integration over proprietary, temperature-only or surface-mounted solutions. The study also highlights technical challenges that remain unresolved, including humidity-sensor durability in highly alkaline environments and increased attenuation in dense or heavily reinforced concrete, which can constrain link margins in demanding deployment conditions.

Accordingly, the present results should be interpreted as evidence of feasibility and early-stage reliability rather than exhaustive long-term validation. Future work will focus on extended multi-year deployments, refined sensor packaging for aggressive chemical environments, and advanced analytics for translating raw multiparameter time series into actionable indicators of structural performance and residual risk.

In summary, ConMonity combines state-of-the-art IoT networks, embedded multi-sensor technology, and cloud analytics in a cost-effective, scalable manner. The platform establishes a foundation for the widespread adoption of intelligent, automated monitoring systems in construction, ultimately enhancing quality, safety, and efficiency throughout the industry.

## Figures and Tables

**Table 1:** A comparison of LoRA and Zigbee sensor node links in construction monitoring context [49].

Zigbee	LoRa	Feature
IEEE 802.15.4	IEEE 802.15.4	Standard
868 MHz, 915 MHz, 2.4 GHz	863 to 870 MHz, 902 to 928 MHz, 915 to 928 MHz, 2.4 GHz	Frequency band
10–100 m per node	>16 km rural, >4.7 km urban	Transmission range
up to 250 kbps (2.4 GHz)	0.3–37.5 kbps	Data rate
20 kbps (868 MHz), 40 Kbps (915 Mhz), 250 Kbps (2.4 GHz)	low	Power consumption
Low, battery life 2–5 years	Ultra-low, battery life 5–10 years	Energy Efficiency
High in mesh/short range	High in rural/wide areas	Reliability
Hundreds of nodes, dense networks	Thousands of nodes, large area	Scalability
Good in mesh, limited in underground	Excellent wall/obstacle penetration	Robustness

**Table 2:** Main functional parameters of ConMonity IoT platform.

Parameter	Value
Strain gauge sensor per node	3
Humidity sensor per node	1
Temperature sensor per node	1
Node depth in concrete, cm	≤20
Node count per gateway	up to 256
Autonomous working time of the node, days	multi-monthly
Autonomous working time of gateway, days	≤7
Battery life per node, years	up to 2

**Table 3:** Comparison of ConMonity with selected commercial concrete monitoring systems.

Feature	ConMonity	Giatec SmartRock (Typical)	Maturix (Typical)
Sensing modalities	Strain, temperature, humidity	Temperature, humidity	Temperature, humidity
Communication (sensor link)	LoRa TDMA	Bluetooth/LoRaWAN	LoRaWAN/NB-IoT
Backhaul	LTE-M	Smartphone/cloud	Cellular/cloud
Depth of embedding	Up to 20 cm	Typically near surface	Typically near surface
Autonomy	Multi-month reported	Vendor-specified	Vendor-specified
Data access	Continuous cloud + alerts	On-site app/cloud	Cloud dashboard
Cost/subscription	COTS components; no licence	Proprietary hardware and app	Proprietary hardware and service

## Data Availability

The data presented in this study are available on request from the corresponding author. The data are not publicly available due to privacy and confidentiality restrictions.
